# Thermophilic bacteria are potential sources of novel Rieske non-heme iron oxygenases

**DOI:** 10.1186/s13568-016-0318-5

**Published:** 2017-01-04

**Authors:** Joydeep Chakraborty, Chiho Suzuki-Minakuchi, Kazunori Okada, Hideaki Nojiri

**Affiliations:** Biotechnology Research Center, The University of Tokyo, 1-1-1 Yayoi, Bunkyo-ku, Tokyo, Japan

**Keywords:** Rieske non-heme iron oxygenase, Oxidoreductase, Thermophiles, Aromatic hydrocarbons, Biotransformation

## Abstract

Rieske non-heme iron oxygenases, which have a Rieske-type [2Fe–2S] cluster and a non-heme catalytic iron center, are an important family of oxidoreductases involved mainly in regio- and stereoselective transformation of a wide array of aromatic hydrocarbons. Though present in all domains of life, the most widely studied Rieske non-heme iron oxygenases are found in mesophilic bacteria. The present study explores the potential for isolating novel Rieske non-heme iron oxygenases from thermophilic sources. Browsing the entire bacterial genome database led to the identification of 45 homologs from thermophilic bacteria distributed mainly among Chloroflexi, Deinococcus–Thermus and Firmicutes. Thermostability, measured according to the aliphatic index, showed higher values for certain homologs compared with their mesophilic relatives. Prediction of substrate preferences indicated that a wide array of aromatic hydrocarbons could be transformed by most of the identified oxygenase homologs. Further identification of putative genes encoding components of a functional oxygenase system opens up the possibility of reconstituting functional thermophilic Rieske non-heme iron oxygenase systems with novel properties.

**Electronic supplementary material:**

The online version of this article (doi:10.1186/s13568-016-0318-5) contains supplementary material, which is available to authorized users.

## Introduction

Rieske non-heme iron oxygenases (ROs) constitute a large family of oxidoreductase enzymes involved primarily in the oxygenation of various aromatic compounds. Although Gibson et al. (1968) first detected the involvement of such an enzyme system in an alkylbenzene-degrading *Pseudomonas* sp., the family has since garnered a great deal of attention for two major reasons. First, ROs are key enzymes responsible for the initial attack on otherwise inert aromatic nuclei, thereby making them targets of a cascade of downstream enzymes, leading to their complete mineralization (Gibson and Subramanian 1984; Allen et al. 1995; Gibson and Parales 2000; Mallick et al. 2011). Secondly, regio- and stereoselective *cis*-dihydroxylation of aromatic compounds, catalyzed by ROs, generate impressive chiral intermediates in the synthesis of a wide array of agrochemically and pharmaceutically important compounds (Ensley et al. 1983; Wackett et al. 1988; Hudlicky et al. 1999; Bui et al. 2002; Newman et al. 2004; Boyd et al. 2005; Zezula and Hudlicky 2005).

Members of the RO family are usually either two- or three-component systems in which one or two soluble electron transport (ET) proteins (such as ferredoxin and reductase) transfer electrons from reduced nucleotides, such as NAD(P)H, to the terminal oxygenase component (a large α-subunit, often accompanied by a small β-subunit),which in turn catalyzes the di- or mono-oxygenation of the aromatic nucleus of the substrate (Mason and Cammack 1992; Ferraro et al. 2005). Numerous ROs have been identified and characterized from bacteria, thereby enriching the available information on their diversity in terms of both sequence and function (Habe and Omori 2003; Iwai et al. 2010, 2011; Chakraborty et al. 2012). Although found in all three domains of life, studies have shown that ROs occur more commonly in bacteria compared with archaea and eukaryotes (Chakraborty et al. 2012). Homologs of the large (α) subunit of RO terminal oxygenase (RO_ox_) have also been investigated in certain plant species, such as *Arabidopsis thaliana*, *Zea mays*, *Pisum sativum*, *Oryza sativa*, *Physcomitrella patens*, *Amaranthus tricolor*, *Ocimum basilicum* and *Spinacia oleracea* (Caliebe et al. 1997; Meng et al. 2001; Reinbothe et al. 2004; Berim et al. 2014), as well as in insects, nematodes and vertebrates (Rottiers et al. 2006; Yoshiyama et al. 2006; Yoshiyama-Yanagawa et al. 2011). ROs from these taxa, however, have entirely different functions from those of bacterial aromatic ring-hydroxylating ROs. They either act as proteintranslocons, facilitating transport across the chloroplastic envelope membranes during chlorophyll biosynthesis, or are involved in flavone and hormone metabolism in plants. They have also been suggested to be involved in regulation of cholesterol metabolism or trafficking during steroid synthesis in insects (Caliebe et al. 1997; Meng et al. 2001; Reinbothe et al. 2004; Rottiers et al. 2006; Yoshiyama et al. 2006; Yoshiyama-Yanagawa et al. 2011; Berim et al. 2014).

Interestingly, few bacterial RO homologs with novel functions, such as oxidative cyclization during biosynthesis of certain antibiotics, hydroxylation and desaturation of short-chain tertiary alcohols and alkane monooxygenation, have been reported in recent years (Sydor et al. 2011; Schäfer et al. 2012; Li et al. 2013). This suggests that ROs bear much more catalytic potential than previously realized. Almost all bacterial ROs characterized biochemically to date have been isolated from mesophilic bacteria, with the sole exception of polychlorinated biphenyl degrading ring-hydroxylating dioxygenase from *Geobacillus* sp. JF8 (Mukerjee-Dhar et al. 2005; Shintani et al. 2014). As such, very little is known about RO homologs present in bacteria that live in extreme environments. Extremophiles, and in particular their enzymes, have proved to be a potentially valuable resource in the development of novel biotechnological processes. The most well-studied extremophiles include thermophiles and hyperthermophiles, and enzymes isolated from such microorganisms are often extremely thermostable and resistant to proteolysis, chemical denaturants, detergents, and organic solvents (Vieille and Zeikus 2001). Apart from enzymatic stability at high temperatures, which is often desired in industrial processes, there are several advantages of thermophilic systems in bioremediation studies. Owing to the poor aqueous solubility of aromatic hydrocarbons, biodegradation studies often encounter problems related to bioavailability. These issues can be overcome at elevated temperatures, since bioavailability tends to increase with temperature owing to increases in solubility (Margesin and Schinner 2001; Feitkenhauer and Märk 2003; Perfumo et al. 2007). Thermophilic microorganisms may thus be attractive candidates for sources of novel thermostable ROs with potential utility in industrial biosynthesis and bioremediation at elevated temperatures.

In recent years, microbial genome sequencing projects have generated an enormous quantity of data for public databases. Since publication of the genome sequence of the first extremophile in 1996 (Bult et al. 1996), there has been a substantial increase in the number of extremophilic genome sequences. Metagenomics and single-cell genomics further add to this repertoire (Hedlund et al. 2014). The present study explored all available genome sequences of thermophilic bacteria for the presence of RO homologs and predicted their suitability as novel RO candidates for biotechnological applications.

## Materials and methods

### Screening thermophilic bacterial genomes for the presence of RO_ox_ α-subunit homologs

Functionally characterized ROs have been categorized into five different similarity classes (A, B, C, D and D*) based on their phylogenetic distribution, substrate preferences and mode of attack on aromatic nuclei (Chakraborty et al. 2012). The National Center for Biotechnology Information (NCBI) ‘genome’ and ‘taxonomy’ databases were searched to characterize the distribution of thermophilic bacteria among different bacterial lineages and the availability of their genome sequences. Representative RO_ox_ α-subunit sequences from each class were used as query probes (Table 1) to perform blastp (Altschul et al. 1990) searches against the translated set of genome sequences. Blast searches were also performed using each thermophilic RO as a query against the NCBI non-redundant database to characterize their distribution among thermophiles (and/or other extremophiles) and mesophiles.

**Table 1:** List of queries used for blast analysis against genomes of thermophilic bacteria

Target gene	Query	Type	Organism	GenBank accession no.
RO α-subunit	Naphthalene dioxygenase (NahAc)	A-IIIαβ	*Pseudomonas putida* NCIB 9816-4	AAO64274
	Benzoate dioxygenase (BenA)	B-IIαβ	*Rhodococcus jostii* RHA1	BAB70698
	Salicylate 5-hydroxylase (NagG)	C-IIIαβ	*Ralstonia* sp. U2	AAD12607
	Carbazole dioxygenase (CarAaII)	D-VIIα	*Sphingomonas* sp. KA1	YP_717942
	3-Ketosteroid 9α-hydroxylase (KshA)	D-Iα	*Mycobacterium tuberculosis* H37rv	NP_218043
Ferredoxin	AntAb associated with anthranilate dioxygenase	Rieske type [2Fe–2S]	*Sphingomonas* sp. KA1	YP_717959
	CarAcI associated with carbazole dioxygenase	Plant type [2Fe–2S]	*Sphingomonas* sp. KA1	YP_717977
	PhtA3 associated with phthalate dioxygenase	[3Fe–4S] type	*Terrabacter* sp. DBF63	BAC54160
	Rub1 associated with naphthalene 1,2-dioxygenase	Rubredoxin	*Rhodococcus* sp. P200	AAR05110
Reductase	KshB associated with 3-Ketosteroid 9α-hydroxylase	FNRc type	*Rhodococcus erythropolis* PR4	BAH32483
	CarAd associated with carbazole dioxygenase	FNRn type	*Pseudomonas* sp. XLDN4-9	AAY56344
	FdrI associated with carbazole dioxygenase	GR type	*Sphingomonas* sp. KA1	YP_718026
	HpaC associated with 4-hydroxyphenylacetate 3-monooxygenase	Flavin reductase	*Thermus thermophilus* HB8	2ECU_A
	Rubredoxin-NAD(+) reductase	Rubredoxin reductase	*Pseudomonas aeruginosa* PAO1	Q9HTK9

*FNRc* ferredoxin-NAD reductase fused with a plant-type [2Fe–2S] domain at the C-terminus,* FNRn* ferredoxin-NAD reductase fused with a plant-type [2Fe–2S] domain at the N-terminus,* GR* glutathione reductase

### Phylogenetic clustering and prediction of substrate preferences

The RHObase server (Chakraborty et al. 2014) was used to categorize each candidate thermophilic RO_ox_ α-subunit into a similarity class and to obtain the closest biochemically characterized homologs. The substrate prediction module of RHObase was further used to predict the substrate preference of the thermophilic homologs and the possible sites of oxygenation. ClustalX v1.81 (Thompson et al. 1997) was used to obtain multiple sequence alignments and to eliminate redundancy among sequences. The default settings were retained for all parameters, with the exception of the matrix (BLOSUM series) used for both pairwise and multiple alignments. Phylogenetic trees were constructed based on distance data using the neighbor-joining method (Saitou and Nei 1987) implemented in ClustalX. The trees were visualized and manipulated using the program TreeExplorer v2.12 (Tamura et al. 2007).

### Verification of the integrity of conserved motifs and domain architecture

The RO_ox_ α-subunit homologs obtained from the genomes of thermophiles were subjected to ScanProsite (De Castro et al. 2006) and NCBI conserved domain database searches (Marchler-Bauer et al. 2002) to verify the presence of conserved sequence motifs. The relevant motifs were C-X-H-X_n_-C-X_2_-H, corresponding to the N-terminal Rieske [2Fe–2S] center, and D-X_2_-H-X_3,4_-H-X_n_-D, corresponding to the C-terminal conserved 2-His-1-carboxylate motif preceded by a conserved aspartate (involved in electron transport), as these are the functional prerequisites of ROs (Jiang et al. 1996; Parales 2003). The motifs were compared with those of phylogenetically close mesophilic ROs. For each protein, the aliphatic index (relative volume occupied by aliphatic side chains) (Ikai 1980) was calculated using ProtParam (Gasteiger et al. 2005).

### Identification of putative ET components

Genomes exhibiting the presence of RO_ox_ α-subunits were searched (using blastp) for genes putatively encoding ET components (both ferredoxin and reductase) using a set of queries (Table 1), followed by manual inspection of each genomic loci when necessary. The queries included the oxidoreductase sequences (e.g., ferredoxin-NAD reductases and glutathione reductase-type reductases and ferredoxins) commonly associated with ROs, as well as other possible oxidoreductases (e.g., flavin reductase and rubredoxin reductase).

## Results

### Distribution of RO homologs among thermophiles

**Fig. 1:**
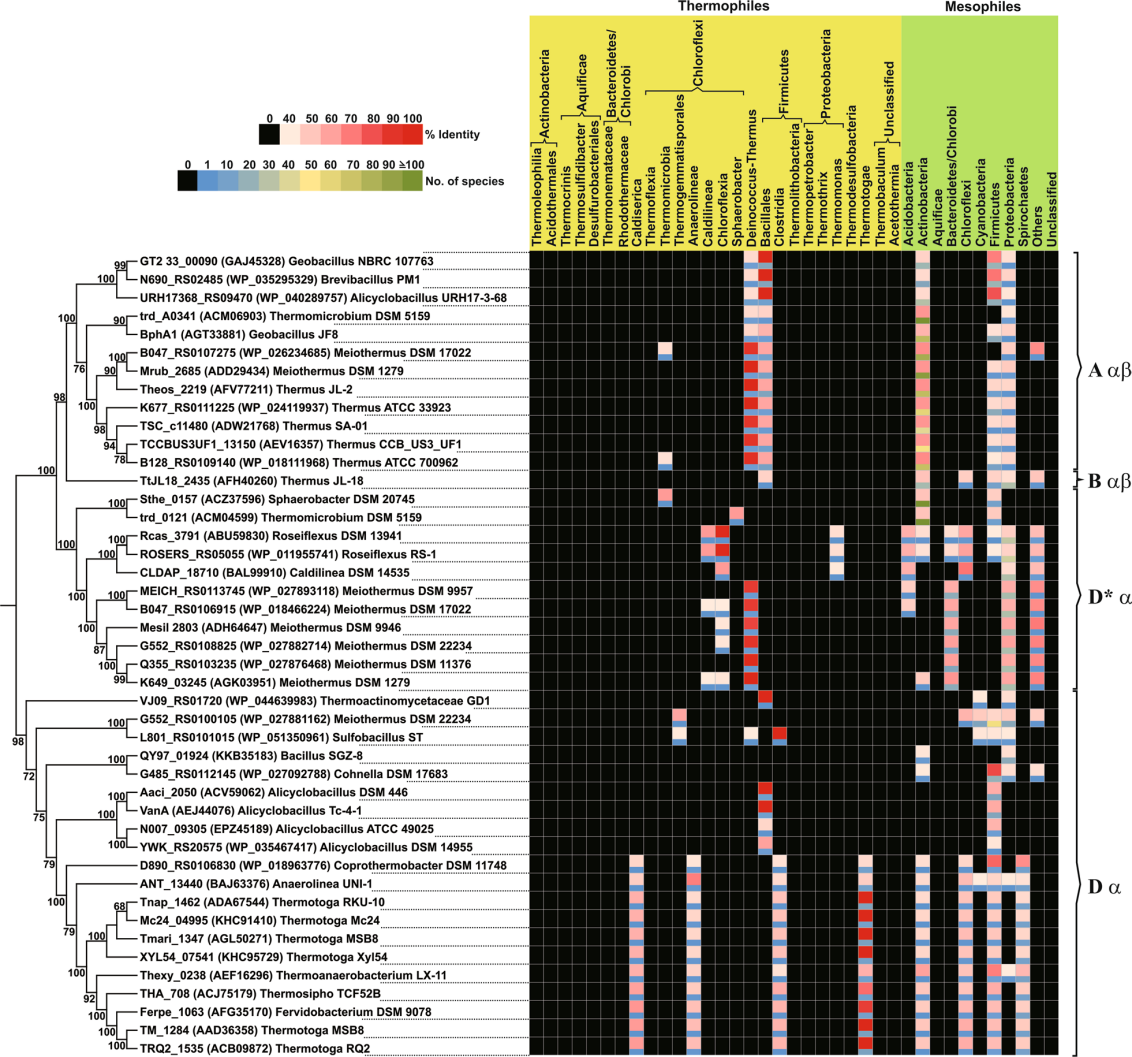
Heat map showing the distribution of α-subunit homologs of thermophilic RO terminal oxygenases among other thermophilic and mesophilic bacteria. Each thermophilic homolog (represented by the corresponding protein name or locus tag followed by the accession number and strain name) was used as a blastp query, and only entries equal to or exceeding the threshold identity of 40% and query coverage of 80% were considered to be positive hits. The distribution is categorized into different taxa (or taxonomic hierarchies), shown on the *top*, with bacteria belonging to each phylum grouped separately as thermophiles and mesophiles (highlighted with *yellow* and *green* backgrounds, respectively). In the heat map, each cell is divided into two blocks; the *upper* (wider) block shows the percentage identity obtained from blastp, while the *lower* (narrower) block indicates the number of distinct species obtained from the blast search. Color codes used for each identity range are shown as an *inset*. Values corresponding to each cell can be obtained from Additional file 1: Table S1

Browsing the bacterial taxonomy database revealed the existence of several thermophilic genera belonging to different classes/orders. These taxa were concentrated mainly among the phyla Thermotogae, Deinococcus–Thermus, Chloroflexi, Aquificae, Firmicutes, and to some extent, Bacteroidetes/Chlorobi, Actinobacteria and Proteobacteria. Blast searches against all thermophile genomes initially led to the identification of 95 putative RO_ox_ α-subunit homologs distributed among 20 different genera (data not shown). Among 45 non-redundant sequences (Table 2), the one obtained from *Alicyclobacillus acidoterrestris* ATCC 49025 (GenBank: EPZ42375) was found to be truncated at the N-terminal end and was therefore excluded from further analysis. Analysis of the distribution of the remaining candidate ROs among both thermophiles and mesophiles revealed that they were present mainly among thermophilic strains belonging to the phyla Chloroflexi, Deinococcus–Thermus, Firmicutes and Thermotogae (Fig. 1). However, distant homologs were abundant among mesophilic strains belonging to the phyla Actinobacteria, Firmicutes and Proteobacteria.

**Table 2:** Putative RO terminal oxygenase α-subunit homologues obtained from blastp search against thermophilic genomes

Organism	Annotation	NCBI accn. no.
Phylum: Chloroflexi		
*Anaerolinea thermophila* UNI-1	Putative oxidoreductase	BAJ63376
*Caldilinea aerophila* DSM 14535	Putative oxidoreductase	BAL99910
*Roseiflexus castenholzii* DSM 13941	Rieske (2Fe–2S) domain protein	ABU59830
*Roseiflexus* sp. RS-1	(2Fe–2S)-binding protein	WP_011955741
*Sphaerobacter thermophilus* DSM 20745	Rieske (2Fe–2S) domain protein	ACZ37596
*Thermomicrobium roseum* DSM 5159	Rieske 2Fe–2S domain protein	ACM04599
*Thermomicrobium roseum* DSM 5159	3-Phenylpropionate (digoxigenin) dioxygenase	ACM06903
Phylum: Deinococcus–Thermus		
*Meiothermus cerbereus* DSM 11376	Choline monooxygenase	WP_027876468
*Meiothermus chliarophilus* DSM 9957	Choline monooxygenase	WP_027893118
*Meiothermus ruber* DSM 1279	Aromatic-ring-hydroxylating dioxygenase, α-subunit	ADD29434
*Meiothermus ruber* DSM 1279	Choline monooxygenase	AGK03951
*Meiothermus rufus* DSM 22234	Hypothetical protein	WP_027881162
*Meiothermus rufus* DSM 22234	Choline monooxygenase	WP_027882714
*Meiothermus silvanus* DSM 9946	Rieske (2Fe–2S) iron–sulfur domain protein	ADH64647
*Meiothermus timidus* DSM 17022	Choline monooxygenase	WP_018466224
*Meiothermus timidus* DSM 17022	Ribosomal subunit interface protein	WP_026234685
*Thermus igniterrae* ATCC 700962	Ribosomal subunit interface protein	WP_018111968
*Thermus oshimai* JL-2	Ring-hydroxylating dioxygenase, large terminal subunit	AFV77211
*Thermus scotoductus* SA-01	Biphenyl dioxygenase, subunit alpha	ADW21768
*Thermus* sp. CCB_US3_UF1	Aromatic-ring-hydroxylating dioxygenase, α-subunit	AEV16357
*Thermus thermophilus* ATCC 33923	Ribosomal subunit interface protein	WP_024119937
*Thermus thermophilus* JL-18	Rieske (2Fe–2S) domain-containing protein	AFH40260
Phylum: Firmicutes		
*Alicyclobacillus acidocaldarius* subsp. *acidocaldarius* DSM 446	Rieske (2Fe–2S) iron–sulphur domain protein	ACV59062
*Alicyclobacillus acidocaldarius* subsp. *acidocaldarius* Tc-4-1	2Fe–2S ferredoxin	AEJ44076
*Alicyclobacillus acidoterrestris* ATCC 49025	Hypothetical protein	EPZ45189
*Alicyclobacillus acidoterrestris* ATCC 49025	Hypothetical protein^a^	EPZ42375
*Alicyclobacillus hesperidum* URH17-3-68	3-Phenylpropionate dioxygenase	WP_040289757
*Alicyclobacillus pomorum* DSM 14955	Hypothetical protein	WP_035467417
*Bacillus thermotolerans* SGZ-8	Phthalate 4,5-dioxygenase oxygenase subunit	KKB35183
*Brevibacillus thermoruber* PM1	3-Phenylpropionate dioxygenase	WP_035295329
*Cohnella thermotolerans* DSM 17683	Rieske (2Fe–2S) protein	WP_027092788
*Coprothermobacter platensis* DSM 11748	Hypothetical protein	WP_018963776
*Geobacillus* sp. JF8	Large subunit of biphenyl dioxygenase	AGT33881
*Geobacillus thermoglucosidasius* NBRC 107763	Putative naphthalene 1,2-dioxygenase large subunit	GAJ45328
*Sulfobacillus thermosulfidooxidans* ST	Hypothetical protein	WP_051350961
Thermoactinomycetaceae bacterium GD1	Rieske (2Fe–2S) protein	WP_044639983
*Thermoanaerobacterium xylanolyticum* LX-11	Rieske (2Fe–2S) iron–sulfur domain protein	AEF16296
Phylum: Thermotogae		
*Fervidobacterium pennivorans* DSM 9078	Ring-hydroxylating dioxygenase, large terminal subunit	AFG35170
*Thermosipho africanus* TCF52B	Oxidase-related protein	ACJ75179
*Thermotoga maritima* MSB8	Oxidase-related protein	AAD36358
*Thermotoga maritima* MSB8	Rieske (2Fe–2S) domain protein	AGL50271
*Thermotoga naphthophila* RKU-10	Rieske (2Fe–2S) iron–sulphur domain protein	ADA67544
*Thermotoga* sp. Mc24	Rieske (2Fe–2S) iron–sulfur domain-containing protein	KHC91410
*Thermotoga* sp. RQ2	Rieske (2Fe–2S) domain protein	ACB09872
*Thermotoga* sp. Xyl54	Rieske (2Fe–2S) iron–sulfur domain-containing protein	KHC95729

^a^Protein truncated at the N-terminal end

### Functional clustering of thermophilic RO_ox_ α-subunit homologs

**Fig. 2:**
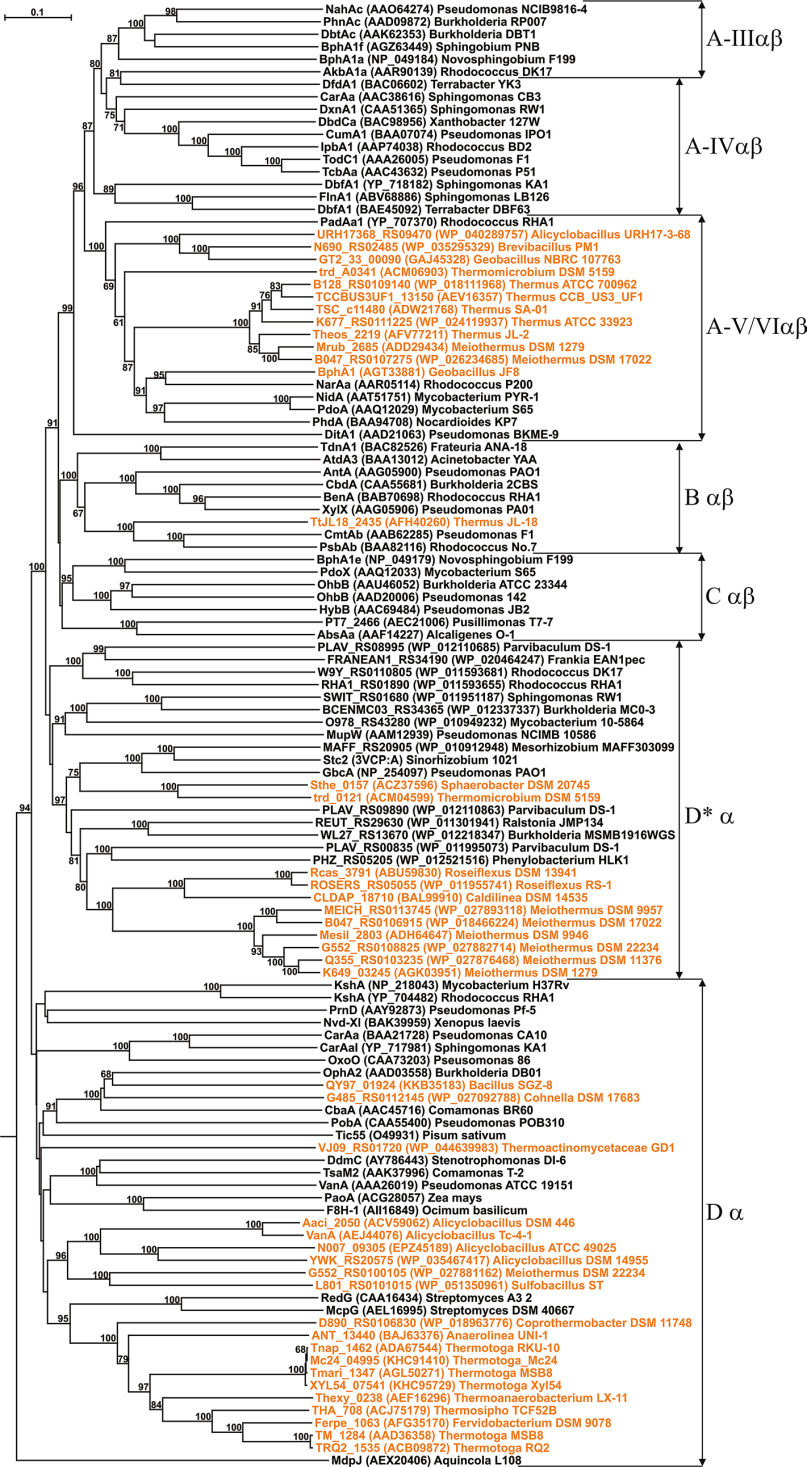
Neighbor-joining tree representing the phylogenetic relationship of putative α-subunits of oxygenase components of ROs obtained from thermophilic bacteria (*orange font*) with homologous sequences from other bacteria and eukaryotes. Each entry is represented by the corresponding protein name or locus tag, followed by the accession number (within *parentheses*) and the strain name. Values at each node indicate the level of bootstrap support based on 100 resampled datasets, while bootstrap values below 60% are not shown. The *bar* represents 0.1 substitutions per amino acid. The sequences have been clustered according to similarity class as defined in Chakraborty et al. (2014)

**Fig. 3:**
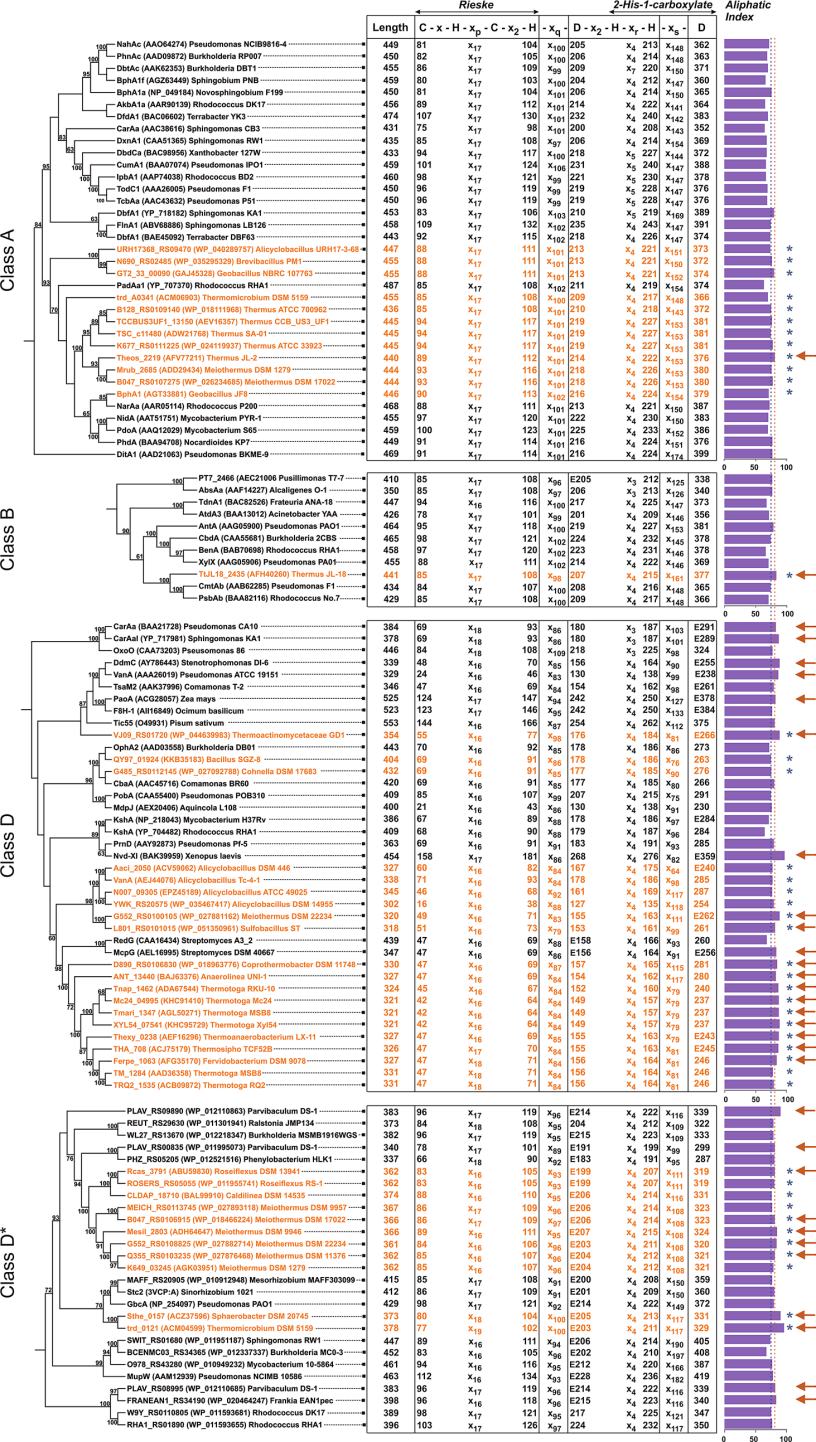
Comparison of conserved N-terminal Rieske [2Fe–2S] and C-terminal 2-His-1-carboxylate motifs among α-subunits of oxygenase components of putative thermophilic ROs obtained from thermophilic bacteria (*orange font*) and those obtained from mesophilic bacteria and eukaryotes. The *horizontal bars* represent the aliphatic index of each sequence. *Blue* and *orange vertical dotted lines* indicate the average aliphatic indices obtained for mesophilic (75.25) and thermophilic (80.88) RO homologs, respectively. All those homologs which showed an aliphatic index ≥80.88 are indicated by an *arrow*, while clades representing only the thermophilic homologs are denoted by *asterisks*

The candidate α-subunit sequences from thermophiles were subjected to phylogenetic studies to assess their relatedness with functionally characterized ROs from other bacteria, as well as from eukaryotes. The phylogenetic tree (Fig. 2) showed that thermophilic homologs were unevenly distributed among all classes of ROs, being clustered in a few specific regions of the tree, again suggesting a radical diversification followed by independent evolution of these genes in thermophiles. As can be seen in Fig. 2, 12 of the sequences clustered with Class A (especially Type A-Vαβ and A-VIαβ) ROs, 1 with Class B ROs, 20 with Class D ROs, and the remaining 11 with Class D* ROs. In all sequences, the conserved N-terminal Rieske [2Fe–2S] motif and C-terminal 2-His-1-carboxylate motif (preceded by a conserved aspartate involved in electron transport), necessary for proper functioning of an RO (Jiang et al. 1996; Parales 2003), is consistent with phylogenetically related, previously characterized ROs (Fig. 3).

The aliphatic index is regarded as a positive factor for the increased thermostability of globular proteins (Ikai 1980). Therefore, we calculated this index for each protein as a measure of thermostability. Not all proteins showed significantly higher values compared with their mesophilic homologs (Fig. 3). However, the average value was found to be higher (80.88) in the thermophilic clusters as compared to those of the mesophiles (75.25), with some proteins (e.g., ROs obtained from Thermoactinomycetaceae bacterium GD1 [GenBank ID WP_044639983], *Meiothermus rufus* DSM 22234 [WP_027881162], *Sphaerobacter thermophilus* DSM 20745 [ACZ37596] and *Thermomicrobium roseum* DSM 5159 [ACM04599]) showing values as high as 90 (Fig. 3).

### Prediction of substrate preferences

**Fig. 4:**
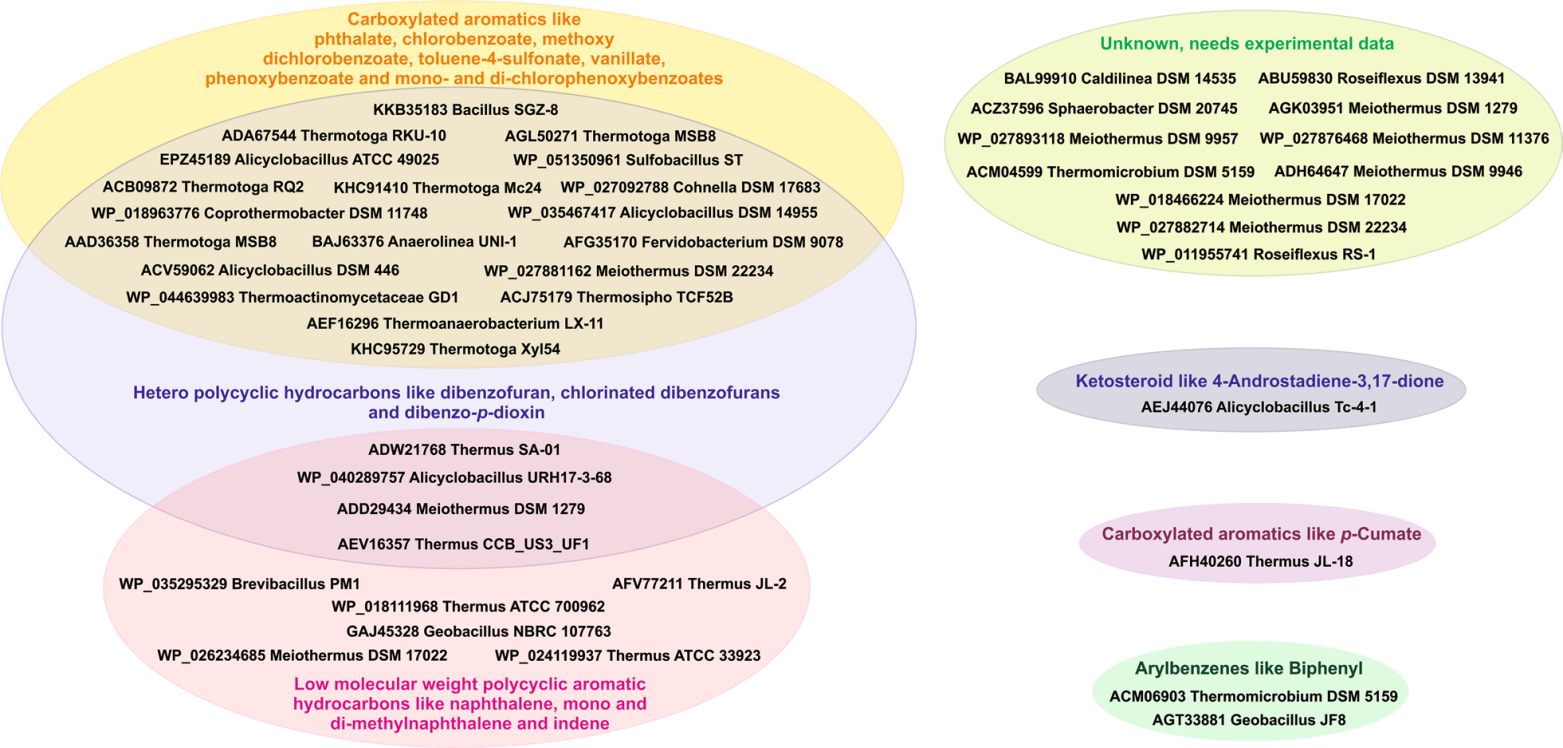
Venn diagram showing the relationship between thermophilic ROs and substrate preference as predicted by RHObase. Putative substrate(s) obtained for each thermophilic RO are shown in detail in Additional file 1: Table S2

Table 3 lists the closest biochemically characterized homolog of each candidate RO_ox_ α-subunit, as obtained from RHObase (Chakraborty et al. 2014). Preferable substrate(s) for most candidate ROs could be predicted using the RHObase substrate prediction module (Fig. 4). RO_ox_ α-subunit homologs belonging to Class A showed a preference for polycyclic aromatic hydrocarbons and heterocyclic polyaromatic hydrocarbons. For one of the Class A ROs, obtained from *Alicyclobacillus acidocaldarius* subsp. *acidocaldarius* Tc-4-1, ketosteroid was found to be its putative substrate. The predicted substrates for the Class B RO_ox_ α-subunit from *Thermus thermophilus* JL-18 were carboxylated aromatics, such as *p*-cumate, while members of Class D showed a preference for carboxylated aromatics such as phthalate, chlorobenzoate, vanillate and phenoxybenzoates, as well as for toluene-4-sulfonate. However, owing to the lack of information regarding the function of Class D* ROs, the substrate preference of these ROs could not be predicted. Apart from MupW and GbcA, involved in the mupirocin (El-Sayed et al. 2003) and glycine betaine (Wargo et al. 2008) biosynthetic pathways, respectively, all other sequences belonging to this class have been derived from whole genome annotations and lack complete information regarding their biochemical function. This makes Class D* the ‘dark matter’ of Rieske oxygenases.

**Table 3:** The closest characterized homologue of each thermophilic RO terminal oxygenase α-subunit sequence obtained from RHObase

Organism (NCBI accession no. of the putative RO terminal oxygenase α-subunit)	Closest match obtained from RHObase	Accession no.	Identity (%)	Query coverage (%)
Class A				
*Thermomicrobium roseum* DSM 5159 (ACM06903)	Biphenyl 2,3-dioxygenase (BphA1) from *Geobacillus* sp. JF8	BAC79226	46.10	91
*Meiothermus ruber* DSM 1279 (ADD29434)	Dibenzofuran dioxygenase (NarAa) from *Rhodococcus opacus* SAO101	BAD02377	50.11	92
*Meiothermus timidus* DSM 17022 (WP_026234685)	Indene dioxygenase (NidA) from *Rhodococcus* sp. I24	AAD25395	49.89	98
*Thermus igniterrae* ATCC 700962 (WP_018111968)	Indene dioxygenase (NidA) from *Rhodococcus* sp. I24	AAD25395	51.48	99
*Thermus oshimai* JL-2 (AFV77211)	Indene dioxygenase (NidA) from *Rhodococcus* sp. I24	AAD25395	50.34	98
*Thermus scotoductus* SA-01 (ADW21768)	Dibenzofuran dioxygenase (NarAa) from *Rhodococcus opacus* SAO101	BAD02377	51.14	99
*Thermus* sp. CCB_US3_UF1 (AEV16357)	Dibenzofuran dioxygenase (NarAa) from *Rhodococcus opacus* SAO101	BAD02377	51.26	97
*Thermus thermophilus* ATCC 33923 (WP_024119937)	Indene dioxygenase (NidA) from *Rhodococcus* sp. I24	AAD25395	51.03	97
*Alicyclobacillus hesperidum* URH17-3-68 (WP_040289757)	Dibenzofuran dioxygenase (NarAa) from *Rhodococcus opacus* SAO101	BAD02377	43.02	93
*Brevibacillus thermoruber* PM1 (WP_035295329)	Indene dioxygenase (NidA) from *Rhodococcus* sp. I24	AAD25395	44.59	92
*Geobacillus* sp. JF8 (AGT33881)	Biphenyl 2,3-dioxygenase (BphA1) from *Geobacillus* sp. JF8	BAC79226	100.00	100
*Geobacillus thermoglucosidasius* NBRC 107763 (GAJ45328)	Indene dioxygenase (NidA) from *Rhodococcus* sp. I24	AAD25395	45.81	93
Class B				
*Thermus thermophilus* JL-18 (AFH40260)	*p*-Cumate dioxygenase (CmtAb) from *Pseudomonas putida* F1	AAB62285	45.37	94
Class D				
*Anaerolinea thermophila* UNI-1 (BAJ63376)	Dicamba *O*-demethylase (DdmC) from *Stenotrophomonas maltophilia* DI-6	AAV53699	32.04	31
*Meiothermus rufus* DSM 22234 (WP_027881162)	Vanillate *O*-demethylase (VanA) from *Pseudomonas* sp. ATCC19151	AAA26019	32.10	47
*Alicyclobacillus acidocaldarius* subsp. *acidocaldarius* DSM 446 (ACV59062)	Toluene-4-sulfonate methyl monooxygenase (TsaM2) from *Comamonas testosteroni* T-2	AAK37996	32.45	32
*Alicyclobacillus acidocaldarius* subsp. *acidocaldarius* Tc-4-1 (AEJ44076)	3-Ketosteroid 9α-hydroxylase (KshA) from *Rhodococcus opacus* B-4	BAH52700	30.54	54
*Alicyclobacillus acidoterrestris* ATCC 49025 (EPZ45189)	Toluene-4-sulfonate methyl monooxygenase (TsaM2) from *Comamonas testosteroni* T-2	AAK37996	30.22	48
*Alicyclobacillus pomorum* DSM 14955 (WP_035467417)	3-Chlorobenzoate-3,4/4,5-dioxygenase (CbaA) from *Comamonas testosteroni* BR60	AAC45716	27.56	50
*Bacillus thermotolerans* SGZ-8 (KKB35183)	Phthalate 4,5-dioxygenase (Pht3) from *Pseudomonas putida*	BAA02511	37.86	94
*Cohnella thermotolerans* DSM 17683 (WP_027092788)	Phthalate 4,5-dioxygenase (OphA2) from *Burkholderia cepacia* DBO1	AAD03558	36.24	97
*Coprothermobacter platensis* DSM 11748 (WP_018963776)	Dicamba *O*-demethylase (DdmC) from *Stenotrophomonas maltophilia* DI-6	AAV53699	32.93	51
*Sulfobacillus thermosulfidooxidans* ST (WP_051350961)	Toluene-4-sulfonate methyl monooxygenase (TsaM2) from *Comamonas testosteroni* T-2	AAK37996	30.12	46
Thermoactinomycetaceae bacterium GD1 (WP_044639983)	Dicamba *O*-demethylase (DdmC) from *Stenotrophomonas maltophilia* DI-6	AAV53699	34.78	54
*Thermoanaerobacterium xylanolyticum* LX-11 (AEF16296)	Vanillate *O*-demethylase (VanA) from *Pseudomonas* sp. HR199	CAA72287	31.84	48
*Fervidobacterium pennivorans* DSM 9078 (AFG35170)	Dicamba *O*-demethylase (DdmC) from *Stenotrophomonas maltophilia* DI-6	AAV53699	34.13	47
*Thermosipho africanus* TCF52B (ACJ75179)	Vanillate *O*-demethylase (VanA) from *Pseudomonas* sp. HR199	CAA72287	33.92	46
*Thermotoga maritima* MSB8 (AAD36358)	3-Chlorobenzoate-3,4/4,5-dioxygenase (CbaA) from *Comamonas testosteroni* BR60	AAC45716	25.35	65
*Thermotoga maritima* MSB8 (AGL50271)	Dicamba *O*-demethylase (DdmC) from *Stenotrophomonas maltophilia* DI-6	AAV53699	32.72	64
*Thermotoga naphthophila* RKU-10 (ADA67544)	Dicamba *O*-demethylase (DdmC) from *Stenotrophomonas maltophilia* DI-6	AAV53699	32.32	73
*Thermotoga* sp. Mc24 (KHC91410)	Dicamba *O*-demethylase (DdmC) from *Stenotrophomonas maltophilia* DI-6	AAV53699	32.72	73
*Thermotoga* sp. RQ2 (ACB09872)	3-Chlorobenzoate-3,4/4,5-dioxygenase (CbaA) from *Comamonas testosteroni* BR60	AAC45716	25.35	65
*Thermotoga* sp. Xyl54 (KHC95729)	Dicamba *O*-demethylase (DdmC) from *Stenotrophomonas maltophilia* DI-6	AAV53699	32.72	73
Class D*				
*Caldilinea aerophila* DSM 14535 (BAL99910)	Phenylpropionate dioxygenase from *Phenylobacterium zucineum* HLK1^a^	WP_012521516	36.50	71
*Roseiflexus castenholzii* DSM 13941(ABU59830)	(2Fe–2S)-binding protein from *Parvibaculum lavamentivorans* DS-1T^a^	WP_011995073	33.92	75
*Roseiflexus* sp. RS-1 (WP_011955741)	(2Fe–2S)-binding protein from *Parvibaculum lavamentivorans* DS-1T^a^	WP_011995074	33.63	81
*Sphaerobacter thermophilus* DSM 20745 (ACZ37596)	Aromatic oxygenase (GbcA) from *Pseudomonas aeruginosa* PAO1	NP_254097	31.36	88
*Thermomicrobium roseum* DSM 5159 (ACM04599)	Aromatic oxygenase (GbcA) from *Pseudomonas aeruginosa* PAO1	NP_254097	29.47	91
*Meiothermus cerbereus* DSM 11376 (WP_027876468)	(2Fe–2S)-binding protein from *Parvibaculum lavamentivorans* DS-1T^a^	WP_011995073	26.91	80
*Meiothermus chliarophilus* DSM 9957 (WP_027893118)	(2Fe–2S)-binding protein from *Parvibaculum lavamentivorans* DS-1T^a^	WP_011995073	28.25	77
*Meiothermus ruber* DSM 1279 (AGK03951)	Rieske (2Fe–2S) domain-containing protein from *Parvibaculum lavamentivorans* DS-1^a^	WP_011995073	40.68	76
*Meiothermus rufus* DSM 22234 (WP_027882714)	Rieske (2Fe–2S) domain-containing protein from *Parvibaculum lavamentivorans* DS-1^a^	WP_011995073	26.84	84
*Meiothermus silvanus* DSM 9946 (ADH64647)	Rieske (2Fe–2S) domain-containing protein from *Parvibaculum lavamentivorans* DS-1^a^	WP_011995073	29.83	80
*Meiothermus timidus* DSM 17022 (WP_018466224)	Rieske (2Fe–2S) domain-containing protein from *Parvibaculum lavamentivorans* DS-1^a^	WP_011995073	27.81	78

^a^Biochemically uncharacterized protein

### Reconstitution of functional RO systems

As discussed earlier, the oxygenase α-subunit is often accompanied by a small β-subunit, and these subunits function in combination with one or two ET component(s). All observations discussed thus far concern the α-subunits of RO_ox_. However, to reconstitute a functional RO system, all of the above components must work together in a coordinated manner. Whenever present, the genes encoding both α-and β-subunits are usually co-localized. Thus, the genome of each thermophile bearing the candidate RO_ox_ α-subunit homologs was screened for the presence of genes putatively encoding the ET component(s). Several putative genes (listed in Table 4) were identified and, in most cases, were located at a distance from the terminal oxygenase genes. As expected, putative ferredoxin and reductase components, along with the β-subunit of RO_ox_, could be identified in most organisms bearing Class A and B ROs. The genome of *Thermus thermophilus* ATCC 33923 had no adjacent β-subunit and did not yield any ferredoxin hits with the queries used. *Alicyclobacillus hesperidum* URH17-3-68 and *Brevibacillus thermoruber* PM1 were also found to lack ferredoxin. Among Class D ROs, D-IVα and D-VIIα type ROs form three-component systems containing both ferredoxin and reductase (Chakraborty et al. 2012). Similarly, putative ferredoxin and reductase components could be identified in several thermophiles bearing Class D ROs. The only exceptions were *Bacillus thermotolerans* SGZ-8 and Thermoactinomycetaceae bacterium GD1, neither of which contained any known component apart from the α-subunit.

**Table 4:** Constituent components, obtained from each thermophile genome, that can be coupled with α-subunit of the terminal oxygenase component in order to reconstitute functional RO system

Organism (NCBI accession no. of the putative RO terminal oxygenase α-subunit)	Protein annotation [accession no.]	Putative function
Class A		
*Thermomicrobium roseum* DSM 5159 (ACM06903)	3-Phenylpropionate dioxygenase subunit beta [ACM06517]	Beta subunit
	Rieske protein [ACM07109] 4Fe–4S ferredoxin, iron–sulfur binding domain protein [ACM06911]	Ferredoxin
	Ferredoxin–NADP reductase; thioredoxin reductase [ACM06977] Putidaredoxin reductase [ACM07176] Thioredoxin reductase [ACM06488]	Reductase
*Meiothermus ruber* DSM 1279 (ADD29434)	Aromatic-ring-hydroxylating dioxygenase beta subunit [ADD29433]	Beta subunit
	Rieske (2Fe–2S) domain protein [ADD27546]	Ferredoxin
	Dihydrolipoamide dehydrogenase [ADD29074]	Reductase
*Meiothermus timidus* DSM 17022 (WP_026234685)	Aromatic-ring-hydroxylating dioxygenase subunit beta [WP_018466297]	Beta subunit
	Diguanylate cyclase [WP_026234838]	Ferredoxin
	Dihydrolipoamide dehydrogenase [WP_026234742] Dihydrolipoamide dehydrogenase [WP_026234716] hypothetical protein [WP_018465799]	Reductase
*Thermus igniterrae* ATCC 700962 (WP_018111968)	Aromatic-ring-hydroxylating dioxygenase subunit beta [WP_018111967]	Beta subunit
*Thermus oshimai* JL-2 (AFV77211)	Small subunit of phenylpropionate dioxygenase [AFV77212]	Beta subunit
	Ferredoxin subunit of nitrite reductase and ring-hydroxylating dioxygenase [AFV75193]	Ferredoxin
	NAD(FAD)-dependent dehydrogenase [AFV75199] Dihydrolipoamide dehydrogenase [AFV77091] Flavoprotein oxygenase [WP_016329217]	Reductase
*Thermus scotoductus* SA-01 (ADW21768)	3-Phenylpropionate dioxygenase, subunit beta [ADW21767]	Beta subunit
	Ferredoxin subunit of phenylpropionate dioxygenase [ADW21026] 4Fe–4S Ferredoxin/formate dehydrogenase, nitrate-inducible, iron–sulfur subunit [ADW21629]	Ferredoxin
	NADH oxidase/coenzyme A disulfide reductase [ADW21032] Phenol hydroxylase component B [ADW21668]	Reductase
*Thermus* sp. CCB_US3_UF1 (AEV16357)	Naphthalene dioxygenase small subunit [AEV16358]	Beta subunit
	Initial dioxygenase ferredoxin subunit [AEV16981]	Ferredoxin
	4-Hydroxybenzoate 3-monooxygenase [AEV16360] FAD-dependent pyridine nucleotide-disulfide oxidoreductase [AEV16975] 4-Hydroxyphenylacetate 3-monooxygenase reductase (Flav_red) [AEV16285]	Reductase
*Thermus thermophilus* ATCC 33923 (WP_024119937)	NADH oxidase [WP_024119527]	Reductase
*Alicyclobacillus hesperidum* URH17-3-68 (WP_040289757)	Thioesterase superfamily protein [EJY55215]	Beta subunit
	Dihydropteridine reductase [WP_006448064] Dihydrolipoamide dehydrogenase [WP_006446492] NADH dehydrogenase [WP_006448257] Thioredoxin reductase [WP_006447474];	Reductase
*Brevibacillus thermoruber* PM1 (WP_035295329)	Aromatic-ring-hydroxylating dioxygenase [WP_035295713]	Beta subunit
	Dihydrolipoamide dehydrogenase [WP_035297436]	Reductase
*Geobacillus* sp. JF8 (AGT33881)	Small subunit of biphenyl dioxygenase [AGT33882]	Beta subunit
	Nitrite reductase NAD(P)H small subunit [AGT32189]	Ferredoxin
	Thioredoxin reductase [AGT33412]	Reductase
*Geobacillus thermoglucosidasius* NBRC 107763 (GAJ45328)	Putative naphthalene 1,2-dioxygenase small subunit [GAJ45329]	Beta subunit
	Hypothetical protein GT2_34_00240 [GAJ45383]	Ferredoxin
	Thioredoxin reductase [GAJ43870] Coenzyme A disulfide reductase [GAJ42754] Putative oxidoreductase [GAJ45381]	Reductase
Class B		
*Thermus thermophilus* JL-18 (AFH40260)	Small subunit of phenylpropionate dioxygenase [AFH40261]	Beta subunit
	Ferredoxin subunit of nitrite reductase and ring-hydroxylating dioxygenase [AFH39800]	Ferredoxin
	NAD(FAD)-dependent dehydrogenase [AFH39794] NAD(FAD)-dependent dehydrogenase [AFH40137]	Reductase
Class D		
*Anaerolinea thermophila* UNI-1 (BAJ63376)	Putative ferredoxin [BAJ63818]	Ferredoxin
	Phytoene dehydrogenase [BAJ63379]	Reductase
*Meiothermus rufus* DSM 22234 (WP_027881162)	Dehydrogenase [WP_027881168]	Reductase
*Alicyclobacillus acidocaldarius* subsp. *acidocaldarius* DSM 446 (ACV59062)	Rieske (2Fe–2S) iron–sulphur domain protein [ACV58029]	Ferredoxin
	Arsenate reductase (thioredoxin) [ACV59045] Flavin reductase domain protein FMN-binding [ACV59470] Flavin reductase domain protein FMN-binding [ACV58921]	Reductase
*Alicyclobacillus acidocaldarius* subsp. *acidocaldarius* Tc-4-1 (AEJ44076)	Rieske (2Fe–2S) domain protein [AEJ42957]	Ferredoxin
	Dihydrolipoamide dehydrogenase [AEJ42545] Flavin reductase domain protein FMN-binding protein [AEJ43919]	Reductase
*Alicyclobacillus acidoterrestris* ATCC 49025 (EPZ45189)	Hypothetical protein N007_08015 [EPZ45711] Hypothetical protein [EPZ45711]	Reductase
*Alicyclobacillus pomorum* DSM 14955 (WP_035467417)	Dihydropteridine reductase [WP_035467306]	Reductase
*Bacillus thermotolerans* SGZ-8 (KKB35183)	Absent	
*Cohnella thermotolerans* DSM 17683 (WP_027092788)	Ferredoxin [WP_027093665]	Ferredoxin
*Coprothermobacter platensis* DSM 11748 (WP_018963776)	2-Enoate reductase [WP_018963737] Hypothetical protein [WP_018963777]	Reductase
*Sulfobacillus thermosulfidooxidans* ST (WP_051350961)	(2Fe–2S)-binding protein [WP_037913512]	Ferredoxin
	Hypothetical protein, partial [WP_040767264]	Reductase
Thermoactinomycetaceae bacterium GD1 (WP_044639983)	Absent	
*Thermoanaerobacterium xylanolyticum* LX-11 (AEF16296)	Rubredoxin-type Fe(Cys)_4_ protein [AEF16485]	Ferredoxin
	Pyruvate ferredoxin/flavodoxin oxidoreductase [AEF16297] CoA-disulfide reductase [AEF16270] Thioredoxin reductase [AEF16306]	Reductase
*Fervidobacterium pennivorans* DSM 9078 (AFG35170)	Rubredoxin [AFG34604]	Ferredoxin
	2-Polyprenylphenol hydroxylase-like oxidoreductase [AFG34955] Phytoene dehydrogenase-like oxidoreductase [AFG35173] Thioredoxin-disulfide reductase [AFG35278] FAD binding protein [AFG35124] Thioredoxin reductase [AFG34984] Dihydrolipoamide dehydrogenase [AFG34941] Thioredoxin reductase [AFG35081]	Reductase
*Thermosipho africanus* TCF52B (ACJ75179)	Rubredoxin [ACJ76024]	Ferredoxin
	Oxidoreductase/NADH: ubiquinone oxidoreductase, na translocating, f-subunit [ACJ75165] Oxidoreductase/tRNA uridine 5-carboxymethylaminomethyl modification enzyme GidA [ACJ75137] Ferredoxin/anaerobic glycerol 3-phosphate dehydrogenase [ACJ75136]	Reductase
*Thermotoga maritima* MSB8 (AAD36358)	Rubredoxin [AGL49584]	Ferredoxin
	Oxidoreductase [AAD35836]	Reductase
*Thermotoga maritima* MSB8 (AGL50271)	Rubredoxin [AGL49584]	Ferredoxin
	Oxidoreductase [AAD35836]	Reductase
*Thermotoga naphthophila* RKU-10 (ADA67544)	Rubredoxin-type Fe(Cys)_4_ protein [ADA66551]	Ferredoxin
	FAD-dependent pyridine nucleotide-disulphide oxidoreductase [ADA67462]	Reductase
*Thermotoga* sp. Mc24 (KHC91410)	Rubredoxin-type Fe(Cys)_4_ protein [KHC90328]	Ferredoxin
	Oxidoreductase [KHC90231]	Reductase
*Thermotoga* sp. RQ2 (ACB09872)	Rubredoxin-type Fe(Cys)_4_ protein [ACB08629]	Ferredoxin
	FAD-dependent pyridine nucleotide-disulphide oxidoreductase [ACB08534]	Reductase
*Thermotoga* sp. Xyl54 (KHC95729)	Rubredoxin-type Fe(Cys)_4_ protein [KHC96420]	Ferredoxin
Class D*		
*Caldilinea aerophila* DSM 14535 (BAL99910)	Hypothetical protein [BAL99023]	Ferredoxin
	Putative mercuric reductase [BAM01871] Putative flavin reductase [BAL99068]	Reductase
*Roseiflexus castenholzii* DSM 13941 (ABU59830)	Rieske (2Fe–2S) domain protein [ABU56444]	Ferredoxin
	Dihydrolipoamide dehydrogenase [ABU58189] Flavin reductase domain protein FMN-binding [ABU60217] Flavin reductase domain protein FMN-binding [ABU58489]	Reductase
*Roseiflexus* sp. RS-1 (WP_011955741)	Rieske (2Fe–2S) domain protein [ABQ89132]	Ferredoxin
	FAD-dependent pyridine nucleotide-disulphide oxidoreductase [ABQ91503] Dihydrolipoamide dehydrogenase [ABQ91367] Flavin reductase domain protein, FMN-binding [ABQ92660] Flavin reductase domain protein, FMN-binding [ABQ90950]	Reductase
*Sphaerobacter thermophilus* DSM 20745 (ACZ37596)	Rieske (2Fe–2S) iron–sulphur domain protein [ACZ40633]	Ferredoxin
	FAD-dependent pyridine nucleotide-disulphide oxidoreductase [ACZ40632] Flavin reductase domain protein FMN-binding protein [ACZ39977]	Reductase
*Thermomicrobium roseum* DSM 5159 (ACM04599)	Rieske protein [ACM07109]	Ferredoxin
	Xylene monooxygenase electron transfer subunit [ACM06651] Ferredoxin–NADP reductase; thioredoxin reductase [ACM06977] Putidaredoxin reductase [ACM07176] Thioredoxin reductase [ACM06488]	Reductase
*Meiothermus cerbereus* DSM 11376 (WP_027876468)	Diguanylatecyclase [WP_027876040]	Ferredoxin
	Dihydrolipoyl dehydrogenase [WP_027876580] MFS transporter [WP_027877386]	Reductase
*Meiothermus chliarophilus* DSM 9957 (WP_027893118)	Dihydrolipoyl dehydrogenase [WP_027893501] MFS transporter [WP_036218580]	Reductase
*Meiothermus ruber* DSM 1279 (AGK03951)	Dihydrolipoamide dehydrogenase [ADD29074] Flavin reductase domain-containing FMN-binding protein [AGK04562]	Reductase
*Meiothermus rufus* DSM 22234 (WP_027882714)	Dehydrogenase [WP_027881168]	Reductase
*Meiothermus silvanus* DSM 9946 (ADH64647)	Rieske (2Fe–2S) iron–sulfur domain protein [ADH62911]	Ferredoxin
	FAD-dependent pyridine nucleotide-disulfide oxidoreductase [ADH64702] Flavin reductase domain protein FMN-binding protein [ADH64172]	Reductase
*Meiothermus timidus* DSM 17022 (WP_018466224)	Dihydrolipoamide dehydrogenase [WP_026234742] Dihydrolipoamide dehydrogenase [WP_026234716] Hypothetical protein [WP_018465799]	Reductase

## Discussion

Owing to their extensive presence among Proteobacteria and Actinobacteria, all extant ROs are postulated to have originated and evolved within these lineages (Chakraborty et al. 2014). Though the thermophilic RO homologs identified in this study were present among taxonomically close organisms, their distributions were very specific for certain phyla, often with very low abundance (Fig. 1). Thus, it is quite likely that the thermophiles also acquired RO_ox_ genes from Proteobacteria or Actinobacteria and further evolved separately. The role of Firmicutes in the evolution of ROs in thermophiles cannot be ruled out, as several RO homologs were found among mesophilic strains of Firmicutes (Fig. 1), especially within the order Bacillales.

Similarly, it would be difficult to claim that only the distantly located ET components identified in this study complement the putative α- and β-subunit genes. It is highly likely that unknown gene(s) vicinal to those encoding the terminal oxygenase(s) are responsible for the proper functioning of the ROs. Similar observations have previously been made in *Rhodococcus opacus* TKN14, in which rubredoxin and another hypothetical protein were found to be crucial for the oxidation of *o*-xylene (Maruyama et al. 2005). In another study, the purified large subunit of a novel alkane monooxygenase (belonging to Class B ROs), identified from a cold-tolerant *Pusillimonas* sp. T7-7, showed NADH-dependent alkane monooxygenase activity (Li et al. 2013). Transformation of aromatic hydrocarbons has also been attained by heterologous expression of the terminal oxygenase components using non-specific ET proteins complemented by the host strain (Mukerjee-Dhar et al. 2005). Although the present study indicates that the RO homologs present in these organisms are either cryptic in nature or are involved in some other physiological function, we cannot rule out the possibility of reconstituting a thermostable functional RO system with novel properties (in terms of substrate preference or mode of catalysis) by combining the terminal oxygenase genes along with all possible combinations of ET components. Integrity of the motif signatures and predicted enhanced thermostability (Fig. 3) further strengthens this hypothesis.

The existence of unexplored microbial diversity, together with the availability of whole genomes, represents a large pool for future industrial catalysts. Thermostable ROs may be attractive candidates for carrying out efficient biotransformation at elevated temperatures. Apart from enhancing our understanding of the distribution of ROs in nature, the present study may aid in designing new bioremediation strategies or industrial biosynthetic processes. Based on the information provided here, functional RO systems can be reconstituted from each organism by cloning both terminal oxygenase and ET genes into suitable vectors and performing biotransformation assays using the predicted substrates (Fig. 4). On the other hand, gene knockout studies can be performed (provided that appropriate genetic tools are available) to help elucidate the physiological role of RO homologs with unknown functions in thermophilic bacteria.

## Additional file

**Additional file 1.** Additional tables.

## Abbreviations

RO: Rieske non-heme iron oxygenase
RO_ox_: large (α) subunit of RO terminal oxygenase
ET: electron transport
NCBI: National Center for Biotechnology Information
NAD(P)H: reduced form of nicotinamide adenine dinucleotide phosphate
