# Supplementary material for: Thermophilic bacteria are potential sources of novel Rieske non-heme iron oxygenases
Source: AMB Express. 2017 Jan 4;7:17. doi: 10.1186/s13568-016-0318-5 (PMC5209329; doi:10.1186/s13568-016-0318-5)
Supplement: Supplementary file 1 — Additional file 1. Additional tables. [file 13568_2016_318_MOESM1_ESM.pdf]

AMB Express
Supporting Information

Thermophilic bacteria are potential sources of novel Rieske non-heme iron oxygenases

Joydeep Chakraborty, Chiho Suzuki-Minakuchi, Kazunori Okada, Hideaki Nojiri*.

Biotechnology Research Center, The University of Tokyo, 1-1-1 Yayoi, Bunkyo-ku, Tokyo, Japan.

***Correspondence:** anojiri@mail.ecc.u-tokyo.ac.jp
Phone & FAX: +81-3-5841-3067

Table S1 Distribution of alpha-subunit homologuesof thermophilic RO terminal oxygenases amongother thermophilic and mesophilic bacteria. Each thermophilic homologue (represented by the corresponding protein name or locus tag followed by accession number and the strain name) was used as blastp query and only entries equal to or above threshold identity of 40% and query coverage 80% were considered as positive hits.The distribution is categorized into different taxa (or taxonomic hierarchies), shown on the top, where bacteria belonging to each phylum have been grouped separately as thermophiles and mesophiles (highlighted with olive and green backgrounds respectively). There are two numbers in each cell where the upper value shows the %identity obtained from blastp while the lower one indicates the number of distinct species obtained from blast search. The heatmap corresponding to this table is shown in Figure 1 in the main text.

	Actinobacteri		Aquificae			Bacteroidetes		Caldise	Chloroflexi							Deinoc	Firmicutes			Proteobacteria			Therm	Therm	Unclassified												
	Therm	Acidot	Therm	Therm	Desulf	Therm	Rhodotherma		Therm	Therm	Therm	Anaer	Caldili	Chloro	Sphaer	Therm	Bacilla	Clostri	Therm	Therm	Therm	Thermomonas			Therm	Acetot	Acidob	Actino	Aquific	Bacter	Chloro	Cyanol	Firmic	Proteo	Spiroc	Other	Unclassified
GT2 33 00090 (GAJ45328) *Geobacillus* NBRC 107763	0	0	0	0	0	0	0	0	0	0	0	0	0	0	0	45	99	0	0	0	0	0	0	0	0	0	0	47	0	0	0	0	69	44	0	0	0
	0	0	0	0	0	0	0	0	0	0	0	0	0	0	0	7	15	0	0	0	0	0	0	0	0	0	0	23	0	0	0	0	21	2	0	0	0
N690 RS02485 (WP 035295329) *Brevibacillus* PM1	0	0	0	0	0	0	0	0	0	0	0	0	0	0	0	45	99	0	0	0	0	0	0	0	0	0	0	45	0	0	0	0	71	47	0	0	0
	0	0	0	0	0	0	0	0	0	0	0	0	0	0	0	3	15	0	0	0	0	0	0	0	0	0	0	15	0	0	0	0	22	2	0	0	0
URH17368 RS09470 (WP 040289757) *Alicyclobacillus* URH17-3-68	0	0	0	0	0	0	0	0	0	0	0	0	0	0	0	44	99	0	0	0	0	0	0	0	0	0	0	45	0	0	0	0	79	42	0	0	0
	0	0	0	0	0	0	0	0	0	0	0	0	0	0	0	8	8	0	0	0	0	0	0	0	0	0	0	29	0	0	0	0	19	2	0	0	0
trd A0341 (ACM06903) *Thermomicrobium* DSM 5159	0	0	0	0	0	0	0	0	0	0	0	0	0	0	0	45	47	0	0	0	0	0	0	0	0	0	0	63	0	0	0	0	0	42	0	0	0
	0	0	0	0	0	0	0	0	0	0	0	0	0	0	0	7	3	0	0	0	0	0	0	0	0	0	0	100	0	0	0	0	0	6	0	0	0
BphA1 (AGT33881) *Geobacillus* JF8	0	0	0	0	0	0	0	0	0	0	0	0	0	0	0	46	55	0	0	0	0	0	0	0	0	0	0	55	0	0	0	0	43	47	0	0	0
	0	0	0	0	0	0	0	0	0	0	0	0	0	0	0	6	6	0	0	0	0	0	0	0	0	0	0	75	0	0	0	0	17	1	0	0	0
B047 RS0107275 (WP 026234685) *Meiothermus* DSM 17022	0	0	0	0	0	0	0	0	0	44	0	0	0	0	0	90	59	0	0	0	0	0	0	0	0	0	0	65	0	0	0	0	0	48	0	66	0
	0	0	0	0	0	0	0	0	0	1	0	0	0	0	0	7	9	0	0	0	0	0	0	0	0	0	0	83	0	0	0	0	0	2	0	2	0
Mrub 2685 (ADD29434) *Meiothermus* DSM 1279	0	0	0	0	0	0	0	0	0	0	0	0	0	0	0	99	58	0	0	0	0	0	0	0	0	0	0	63	0	0	0	0	45	49	0	0	0
	0	0	0	0	0	0	0	0	0	0	0	0	0	0	0	7	11	0	0	0	0	0	0	0	0	0	0	94	0	0	0	0	3	5	0	0	0
Theos 2219 (AFV77211) *Thermus* JL-2	0	0	0	0	0	0	0	0	0	0	0	0	0	0	0	99	59	0	0	0	0	0	0	0	0	0	0	64	0	0	0	0	46	46	0	0	0
	0	0	0	0	0	0	0	0	0	0	0	0	0	0	0	7	14	0	0	0	0	0	0	0	0	0	0	74	0	0	0	0	8	2	0	0	0
K677 RS0111225 (WP 024119937) *Thermus* ATCC 33923	0	0	0	0	0	0	0	0	0	0	0	0	0	0	0	99	59	0	0	0	0	0	0	0	0	0	0	63	0	0	0	0	45	47	0	0	0
	0	0	0	0	0	0	0	0	0	0	0	0	0	0	0	7	7	0	0	0	0	0	0	0	0	0	0	60	0	0	0	0	17	2	0	0	0
TSC c11480 (ADW21768) *Thermus* SA-01	0	0	0	0	0	0	0	0	0	0	0	0	0	0	0	89	59	0	0	0	0	0	0	0	0	0	0	63	0	0	0	0	45	47	0	0	0
	0	0	0	0	0	0	0	0	0	0	0	0	0	0	0	8	5	0	0	0	0	0	0	0	0	0	0	42	0	0	0	0	2	3	0	0	0
TCCBUS3UF1 13150 (AEV16357) *Thermus* CCB US3 UF1	0	0	0	0	0	0	0	0	0	0	0	0	0	0	0	90	59	0	0	0	0	0	0	0	0	0	0	63	0	0	0	0	44	47	0	0	0
	0	0	0	0	0	0	0	0	0	0	0	0	0	0	0	6	9	0	0	0	0	0	0	0	0	0	0	53	0	0	0	0	9	3	0	0	0
B128 RS0109140 (WP 018111968) *Thermus* ATCC 700962	0	0	0	0	0	0	0	0	0	43	0	0	0	0	0	91	59	0	0	0	0	0	0	0	0	0	0	66	0	0	0	0	41	46	0	0	0
	0	0	0	0	0	0	0	0	0	1	0	0	0	0	0	7	11	0	0	0	0	0	0	0	0	0	0	72	0	0	0	0	11	1	0	0	0
TtJL18 2435 (AFH40260) *Thermus* JL-18	0	0	0	0	0	0	0	0	0	0	0	0	0	0	0	0	47	0	0	0	0	0	0	0	0	0	0	51	0	0	48	0	47	44	0	50	0
	0	0	0	0	0	0	0	0	0	0	0	0	0	0	0	0	1	0	0	0	0	0	0	0	0	0	0	25	0	0	1	0	10	28	0	2	0
Sthe 0157 (ACZ37596) *Sphaerobacter* DSM 20745	0	0	0	0	0	0	0	0	0	62	0	0	0	0	0	0	0	0	0	0	0	0	0	0	0	0	0	51	0	0	0	0	48	0	0	0	0
	0	0	0	0	0	0	0	0	0	1	0	0	0	0	0	0	0	0	0	0	0	0	0	0	0	0	0	200	0	0	0	0	10	0	0	0	0
trd 0121 (ACM04599) *Thermomicrobium* DSM 5159	0	0	0	0	0	0	0	0	0	0	0	0	0	0	62	0	0	0	0	0	0	0	0	0	0	0	0	51	0	0	0	0	49	0	0	0	0
	0	0	0	0	0	0	0	0	0	0	0	0	0	0	1	0	0	0	0	0	0	0	0	0	0	0	0	200	0	0	0	0	8	0	0	0	0
Rcas 3791 (ABU59830) *Roseiflexus* DSM 13941	0	0	0	0	0	0	0	0	0	0	0	0	62	95	0	0	0	0	0	0	0	44	0	0	0	0	56	44	0	44	59	0	40	48	0	53	0
	0	0	0	0	0	0	0	0	0	0	0	0	1	1	0	0	0	0	0	0	0	1	0	0	0	0	3	3	0	1	3	0	1	32	0	5	0
ROSERS RS05055 (WP) 011955741 *Roseiflexus* RS-1	0	0	0	0	0	0	0	0	0	0	0	0	62	95	0	0	0	0	0	0	0	44	0	0	0	0	56	43	0	45	60	0	40	47	0	54	0
	0	0	0	0	0	0	0	0	0	0	0	0	1	1	0	0	0	0	0	0	0	1	0	0	0	0	3	3	0	1	3	0	1	36	0	5	0
CLDAP 18710 (BAL99910) *Caldilinea* DSM 14535	0	0	0	0	0	0	0	0	0	0	0	0	0	62	0	0	0	0	0	0	0	41	0	0	0	0	56	0	0	0	71	0	0	45	0	53	0
	0	0	0	0	0	0	0	0	0	0	0	0	0	2	0	0	0	0	0	0	0	1	0	0	0	0	3	0	0	0	2	0	0	28	0	5	0
MEICH RS0113745 (WP 027893118) *Meiothermus* DSM 9957	0	0	0	0	0	0	0	0	0	0	0	0	0	0	0	87	0	0	0	0	0	0	0	0	0	0	48	0	0	57	0	0	0	57	0	65	0
	0	0	0	0	0	0	0	0	0	0	0	0	0	0	0	5	0	0	0	0	0	0	0	0	0	0	1	0	0	18	0	0	0	26	0	2	0
B047 RS0106915 (WP 018466224) *Meiothermus* DSM 17022	0	0	0	0	0	0	0	0	0	0	0	0	40	40	0	87	0	0	0	0	0	0	0	0	0	0	48	0	0	59	0	0	0	56	0	66	0
	0	0	0	0	0	0	0	0	0	0	0	0	1	2	0	5	0	0	0	0	0	0	0	0	0	0	1	0	0	18	0	0	0	28	0	2	0
Mesil 2803 (ADH64647) *Meiothermus* DSM 9946	0	0	0	0	0	0	0	0	0	0	0	0	0	42	0	84	0	0	0	0	0	0	0	0	0	0	0	0	0	59	0	0	0	59	0	70	0
	0	0	0	0	0	0	0	0	0	0	0	0	0	2	0	5	0	0	0	0	0	0	0	0	0	0	0	0	0	17	0	0	0	25	0	2	0
G552 RS0108825 (WP 027882714) *Meiothermus* DSM 22234	0	0	0	0	0	0	0	0	0	0	0	0	0	42	0	90	0	0	0	0	0	0	0	0	0	0	0	0	0	61	0	0	0	60	0	68	0

	0	0	0	0	0	0	0	0	0	0	0	0	0	2	0	5	0	0	0	0	0	0	0	0	0	0	0	0	0	12	0	0	0	27	0	1	0
Q355 RS0103235 (WP 027876468) *Meiothermus* DSM 11376	0	0	0	0	0	0	0	0	0	0	0	0	0	0	0	94	0	0	0	0	0	0	0	0	0	0	0	0	0	61	0	0	0	58	0	67	0
	0	0	0	0	0	0	0	0	0	0	0	0	0	0	0	5	0	0	0	0	0	0	0	0	0	0	0	0	0	15	0	0	0	27	0	2	0
K649 03245 (AGK03951) *Meiothermus* DSM 1279	0	0	0	0	0	0	0	0	0	0	0	0	40	42	0	99	0	0	0	0	0	0	0	0	0	0	0	49	0	63	0	0	0	59	0	68	0
	0	0	0	0	0	0	0	0	0	0	0	0	1	2	0	6	0	0	0	0	0	0	0	0	0	0	0	1	0	17	0	0	0	27	0	2	0
VJ09 RS01720 (WP 044639983) *Thermoactinomycetaceae* GD1	0	0	0	0	0	0	0	0	0	0	0	0	0	0	0	0	100	0	0	0	0	0	0	0	0	0	0	0	0	0	0	41	0	44	0	0	0
	0	0	0	0	0	0	0	0	0	0	0	0	0	0	0	0	1	0	0	0	0	0	0	0	0	0	0	0	0	0	0	2	0	1	0	0	0
G552 RS0100105 (WP 027881162) *Meiothermus* DSM 22234	0	0	0	0	0	0	0	0	0	0	59	0	0	0	0	0	0	0	0	0	0	0	0	0	0	0	0	0	0	0	55	49	46	49	0	48	0
	0	0	0	0	0	0	0	0	0	0	1	0	0	0	0	0	0	0	0	0	0	0	0	0	0	0	0	0	0	0	1	14	55	16	0	2	0
L801 RS0101015 (WP 051350961) *Sulfobacillus* ST	0	0	0	0	0	0	0	0	0	0	41	0	0	0	0	40	0	99	0	0	0	0	0	0	0	0	0	0	0	0	0	42	42	41	0	0	0
	0	0	0	0	0	0	0	0	0	0	1	0	0	0	0	1	0	1	0	0	0	0	0	0	0	0	0	0	0	0	0	4	1	4	0	0	0
QY97 01924 (KKB35183) *Bacillus* SGZ-8	0	0	0	0	0	0	0	0	0	0	0	0	0	0	0	0	0	0	0	0	0	0	0	0	0	0	0	42	0	0	0	0	0	44	0	0	0
	0	0	0	0	0	0	0	0	0	0	0	0	0	0	0	0	0	0	0	0	0	0	0	0	0	0	0	5	0	0	0	0	0	17	0	0	0
G485 RS0112145 (WP 027092788) *Cohnella* DSM 17683	0	0	0	0	0	0	0	0	0	0	0	0	0	0	0	0	0	0	0	0	0	0	0	0	0	0	0	41	0	0	0	0	82	44	0	44	0
	0	0	0	0	0	0	0	0	0	0	0	0	0	0	0	0	0	0	0	0	0	0	0	0	0	0	0	2	0	0	0	0	20	9	0	1	0
Aaci 2050 (ACV59062) *Alicyclobacillus* DSM 446	0	0	0	0	0	0	0	0	0	0	0	0	0	0	0	0	99	0	0	0	0	0	0	0	0	0	0	0	0	0	0	0	57	0	0	0	0
	0	0	0	0	0	0	0	0	0	0	0	0	0	0	0	0	8	0	0	0	0	0	0	0	0	0	0	0	0	0	0	0	14	0	0	0	0
VanA (AEJ44076) *Alicyclobacillus* Tc-4-1	0	0	0	0	0	0	0	0	0	0	0	0	0	0	0	0	99	0	0	0	0	0	0	0	0	0	0	0	0	0	0	0	58	0	0	0	0
	0	0	0	0	0	0	0	0	0	0	0	0	0	0	0	0	8	0	0	0	0	0	0	0	0	0	0	0	0	0	0	0	15	0	0	0	0
N007 09305 (EPZ45189) *Alicyclobacillus* ATCC 49025	0	0	0	0	0	0	0	0	0	0	0	0	0	0	0	0	46	0	0	0	0	0	0	0	0	0	0	0	0	0	0	0	55	0	0	0	0
	0	0	0	0	0	0	0	0	0	0	0	0	0	0	0	0	1	0	0	0	0	0	0	0	0	0	0	0	0	0	0	0	6	0	0	0	0
YWK RS20575 (WP 035467417) *Alicyclobacillus* DSM 14955	0	0	0	0	0	0	0	0	0	0	0	0	0	0	0	0	60	0	0	0	0	0	0	0	0	0	0	0	0	0	0	0	42	0	0	0	0
	0	0	0	0	0	0	0	0	0	0	0	0	0	0	0	0	6	0	0	0	0	0	0	0	0	0	0	0	0	0	0	0	1	0	0	0	0
D890 RS0106830 (WP 018963776) *Coprothermobacter* DSM 11748	0	0	0	0	0	0	0	46	0	0	0	42	0	0	0	0	0	43	0	0	0	0	0	45	0	0	0	44	0	0	46	0	75	0	65	0	0
	0	0	0	0	0	0	0	1	0	0	0	4	0	0	0	0	0	1	0	0	0	0	0	9	0	0	0	1	0	0	2	0	7	0	9	0	0
ANT 13440 (BAJ63376) *Anaerolinea* UNI-1	0	0	0	0	0	0	0	48	0	0	0	69	0	0	0	0	0	45	0	0	0	0	0	48	0	0	0	45	0	0	53	35	46	41	43	0	0
	0	0	0	0	0	0	0	1	0	0	0	3	0	0	0	0	0	2	0	0	0	0	0	10	0	0	0	1	0	0	2	2	7	1	9	0	0
Tnap 1462 (ADA67544) *Thermotoga* RKU-10	0	0	0	0	0	0	0	56	0	0	0	45	0	0	0	0	0	51	0	0	0	0	0	99	0	0	0	45	0	0	51	0	49	0	45	0	0
	0	0	0	0	0	0	0	1	0	0	0	2	0	0	0	0	0	2	0	0	0	0	0	14	0	0	0	1	0	0	3	0	3	0	10	0	0
Mc24 04995 (KHC91410) *Thermotoga* Mc24	0	0	0	0	0	0	0	56	0	0	0	46	0	0	0	0	0	52	0	0	0	0	0	100	0	0	0	46	0	0	51	0	49	0	46	0	0
	0	0	0	0	0	0	0	1	0	0	0	3	0	0	0	0	0	2	0	0	0	0	0	10	0	0	0	1	0	0	3	0	3	0	10	0	0
Tmari 1347 (AGL50271) *Thermotoga* MSB8	0	0	0	0	0	0	0	56	0	0	0	46	0	0	0	0	0	52	0	0	0	0	0	100	0	0	0	46	0	0	53	0	50	0	46	0	0
	0	0	0	0	0	0	0	1	0	0	0	2	0	0	0	0	0	1	0	0	0	0	0	12	0	0	0	1	0	0	2	0	4	0	9	0	0
XYL54 07541 (KHC95729) *Thermotoga* Xyl54	0	0	0	0	0	0	0	56	0	0	0	46	0	0	0	0	0	52	0	0	0	0	0	100	0	0	0	46	0	0	51	0	49	0	46	0	0
	0	0	0	0	0	0	0	1	0	0	0	3	0	0	0	0	0	2	0	0	0	0	0	10	0	0	0	1	0	0	3	0	3	0	10	0	0
Thexy 0238 (AEF16296) *Thermoanaerobacterium* LX-11	0	0	0	0	0	0	0	56	0	0	0	47	0	0	0	0	0	43	0	0	0	0	0	56	0	0	0	60	0	0	51	0	69	40	54	0	0
	0	0	0	0	0	0	0	1	0	0	0	3	0	0	0	0	0	1	0	0	0	0	0	8	0	0	0	1	0	0	3	0	7	1	11	0	0
THA 708 (ACJ75179) *Thermosipho* TCF52B	0	0	0	0	0	0	0	60	0	0	0	49	0	0	0	0	0	57	0	0	0	0	0	73	0	0	0	50	0	0	51	0	57	0	49	0	0
	0	0	0	0	0	0	0	1	0	0	0	3	0	0	0	0	0	2	0	0	0	0	0	8	0	0	0	1	0	0	3	0	5	0	10	0	0
Ferpe 1063 (AFG35170) *Fervidobacterium* DSM 9078	0	0	0	0	0	0	0	63	0	0	0	52	0	0	0	0	0	55	0	0	0	0	0	86	0	0	0	49	0	0	54	0	53	0	48	0	0
	0	0	0	0	0	0	0	1	0	0	0	3	0	0	0	0	0	2	0	0	0	0	0	10	0	0	0	1	0	0	3	0	7	0	11	0	0
TM_1284 (AAD36358) *Thermotoga* MSB8	0	0	0	0	0	0	0	62	0	0	0	53	0	0	0	0	0	55	0	0	0	0	0	99	0	0	0	50	0	0	57	0	53	0	49	0	0
	0	0	0	0	0	0	0	1	0	0	0	2	0	0	0	0	0	2	0	0	0	0	0	12	0	0	0	1	0	0	3	0	6	0	10	0	0
TRQ2_1535 (ACB09872) *Thermotoga* RQ2	0	0	0	0	0	0	0	62	0	0	0	53	0	0	0	0	0	55	0	0	0	0	0	99	0	0	0	50	0	0	57	0	53	0	49	0	0
	0	0	0	0	0	0	0	1	0	0	0	3	0	0	0	0	0	2	0	0	0	0	0	12	0	0	0	1	0	0	3	0	6	0	10	0	0

Table S2 Substrate preference of thermophilic ROs predicted using RHObase

**Organism (NCBI accession no. of the putative α-subunit of RO oxygenase component)**	**Putative substrate**
**Class A**	
Thermomicrobium roseum DSM 5159(ACM06903)	Arylbenzenes like biphenyl
Meiothermus ruber DSM 1279(ADD29434)	Low molecular weight polycyclic aromatic hydrocarbons like naphthalene, mono and di-methylnaphthalene and indene, Hetero polycyclic hydrocarbons like dibenzofuran and dibenzo-*p*-dioxin
Meiothermus timidus DSM 17022(WP_026234685)	Low molecular weight polycyclic aromatic hydrocarbons like naphthalene and indene
Thermus igniterrae ATCC 700962 (WP_018111968)	Low molecular weight polycyclic aromatic hydrocarbons like naphthalene and indene
Thermusoshimai JL-2 (AFV77211)	Low molecular weight polycyclic aromatic hydrocarbons like naphthalene and indene
Thermusscotoductus SA-01 (ADW21768)	Low molecular weight polycyclic aromatic hydrocarbons like naphthalene, mono and di-methylnaphthalene and indene, Hetero polycyclic hydrocarbons like dibenzofuran and dibenzo-*p*-dioxin
Thermus sp. CCB_US3_UF1(AEV16357)	Low molecular weight polycyclic aromatic hydrocarbons like naphthalene, mono and di-methylnaphthalene and indene, Hetero polycyclic hydrocarbons like dibenzofuran and dibenzo-*p*-dioxin
Thermusthermophilus ATCC 33923 (WP_024119937)	Low molecular weight polycyclic aromatic hydrocarbons like naphthalene and indene
Alicyclobacillushesperidum URH17-3-68 (WP_040289757)	Low molecular weight polycyclic aromatic hydrocarbons like naphthalene, mono and di-methylnaphthalene and Indene, Hetero polycyclic hydrocarbons like dibenzofuran and dibenzo-*p*-dioxin
Brevibacillus thermoruber PM1 (WP_035295329)	Low molecular weight polycyclic aromatic hydrocarbons like naphthalene and indene
Geobacillus sp. JF8 (AGT33881)	Biphenyl
Geobacillusthermoglucosidasius NBRC 107763 (GAJ45328)	Low molecular weight polycyclic aromatic hydrocarbons like naphthalene and indene
**Class B**	
Thermusthermophilus JL-18 (AFH40260)	Carboxylated aromatics like *p*-cumate
**Class D**	
Anaerolinea thermophila UNI-1 (BAJ63376)	Carboxylated aromatics like phthalate, chlorobenzoate, methoxy dichlorobenzoate, toluene-4-sulfonate, vanillate, phenoxybenzoate and mono- and di-chlorophenoxybenzoates, hetero polycyclic hydrocarbons like chlorinated dibenzofurans
Meiothermus rufus DSM 22234	Carboxylated aromatics like phthalate, chlorobenzoate, methoxy dichlorobenzoate, toluene-4-sulfonate, vanillate,

(WP_027881162)	phenoxybenzoate and mono- and di-chlorophenoxybenzoates, hetero polycyclic hydrocarbons like chlorinated dibenzofurans
Alicyclobacillus acidocaldarius subsp. *acidocaldarius* DSM 446 (ACV59062)	Carboxylated aromatics like phthalate, chlorobenzoate, methoxy dichlorobenzoate, toluene-4-sulfonate, vanillate, phenoxybenzoate and mono- and di-chlorophenoxybenzoates, hetero polycyclic hydrocarbons like chlorinated dibenzofurans
Alicyclobacillus acidocaldarius subsp. *acidocaldarius* Tc-4-1(AEJ44076)	Ketosteroid like 4-androstadiene-3,17-dione
Alicyclobacillusacidoterrestris ATCC 49025(EPZ45189)	Carboxylated aromatics like phthalate, chlorobenzoate, methoxy dichlorobenzoate, toluene-4-sulfonate, vanillate, phenoxybenzoate and mono- and di-chlorophenoxybenzoates, hetero polycyclic hydrocarbons like chlorinated dibenzofurans
Alicyclobacillus pomorum DSM 14955(WP_035467417)	Carboxylated aromatics like phthalate, chlorobenzoate, methoxy dichlorobenzoate, toluene-4-sulfonate, vanillate, phenoxybenzoate and mono- and di-chlorophenoxybenzoates, hetero polycyclic hydrocarbons like chlorinated dibenzofurans
Bacillus thermotolerans SGZ-8 (KKB35183)	Carboxylated aromatics like phthalate, chlorobenzoate, methoxy dichlorobenzoate, toluene-4-sulfonate, vanillate, phenoxybenzoate and mono- and di-chlorophenoxybenzoates, hetero polycyclic hydrocarbons like chlorinated dibenzofurans
Cohnella thermotolerans DSM 17683(WP_027092788)	Carboxylated aromatics like phthalate, chlorobenzoate, methoxy dichlorobenzoate, toluene-4-sulfonate, vanillate, phenoxybenzoate and mono- and di-chlorophenoxybenzoates, hetero polycyclic hydrocarbons like chlorinated dibenzofurans
Coprothermobacter platensis DSM 11748(WP_018963776)	Carboxylated aromatics like phthalate, chlorobenzoate, methoxy dichlorobenzoate, toluene-4-sulfonate, vanillate, phenoxybenzoate and mono- and di-chlorophenoxybenzoates, hetero polycyclic hydrocarbons like chlorinated dibenzofurans
*Sulfobacillus thermosulfidooxidans*ST(WP_051350961)	Carboxylated aromatics like phthalate, chlorobenzoate, methoxy dichlorobenzoate, toluene-4-sulfonate, vanillate, phenoxybenzoate and mono- and di-chlorophenoxybenzoates, hetero polycyclic hydrocarbons like chlorinated dibenzofurans
Thermoactinomycetaceae bacterium GD1(WP_044639983)	Carboxylated aromatics like phthalate, chlorobenzoate, methoxy dichlorobenzoate, toluene-4-sulfonate, vanillate, phenoxybenzoate and mono- and di-chlorophenoxybenzoates, hetero polycyclic hydrocarbons like chlorinated dibenzofurans
Thermoanaerobacteriumxylanolyticum LX-11(AEF16296)	Carboxylated aromatics like phthalate, chlorobenzoate, methoxy dichlorobenzoate, toluene-4-sulfonate, vanillate, phenoxybenzoate and mono- and di-chlorophenoxybenzoates, hetero polycyclic hydrocarbons like chlorinated dibenzofurans
Fervidobacteriumpennivorans DSM 9078(AFG35170)	Carboxylated aromatics like phthalate, chlorobenzoate, methoxy dichlorobenzoate, toluene-4-sulfonate, vanillate, phenoxybenzoate and mono- and di-chlorophenoxybenzoates, hetero polycyclic hydrocarbons like chlorinated dibenzofurans
Thermosiphoafricanus TCF52B(ACJ75179)	Carboxylated aromatics like phthalate, chlorobenzoate, methoxy dichlorobenzoate, toluene-4-sulfonate, vanillate, phenoxybenzoate and mono- and di-chlorophenoxybenzoates, hetero polycyclic hydrocarbons like chlorinated dibenzofurans

Thermotogamaritima MSB8(AAD36358)	Carboxylated aromatics like phthalate, chlorobenzoate, methoxy dichlorobenzoate, toluene-4-sulfonate, vanillate, phenoxybenzoate and mono- and di-chlorophenoxybenzoates, hetero polycyclic hydrocarbons like chlorinated dibenzofurans
Thermotogamaritima MSB8(AGL50271)	Carboxylated aromatics like phthalate, chlorobenzoate, methoxy dichlorobenzoate, toluene-4-sulfonate, vanillate, phenoxybenzoate and mono- and di-chlorophenoxybenzoates, hetero polycyclic hydrocarbons like chlorinated dibenzofurans
Thermotoganaphthophila RKU-10(ADA67544)	Carboxylated aromatics like phthalate, chlorobenzoate, methoxy dichlorobenzoate, toluene-4-sulfonate, vanillate, phenoxybenzoate and mono- and di-chlorophenoxybenzoates, hetero polycyclic hydrocarbons like chlorinated dibenzofurans
Thermotoga sp. Mc24(KHC91410)	Carboxylated aromatics like phthalate, chlorobenzoate, methoxy dichlorobenzoate, toluene-4-sulfonate, vanillate, phenoxybenzoate and mono- and di-chlorophenoxybenzoates, hetero polycyclic hydrocarbons like chlorinated dibenzofurans
Thermotoga sp. RQ2(ACB09872)	Carboxylated aromatics like phthalate, chlorobenzoate, methoxy dichlorobenzoate, toluene-4-sulfonate, vanillate, phenoxybenzoate and mono- and di-chlorophenoxybenzoates, hetero polycyclic hydrocarbons like chlorinated dibenzofurans
Thermotoga sp. Xyl54(KHC95729)	Carboxylated aromatics like phthalate, chlorobenzoate, methoxy dichlorobenzoate, toluene-4-sulfonate, vanillate, phenoxybenzoate and mono- and di-chlorophenoxybenzoates, hetero polycyclic hydrocarbons like chlorinated dibenzofurans
**Class D***	
Caldilinea aerophila DSM 14535(BAL99910)	Unknown, needs experimental data
Roseiflexus castenholzii DSM 13941(ABU59830)	Unknown, needs experimental data
Roseiflexus sp. RS-1(WP_011955741)	Unknown, needs experimental data
Sphaerobacter thermophilus DSM 20745(ACZ37596)	Unknown, needs experimental data
Thermomicrobium roseum DSM 5159(ACM04599)	Unknown, needs experimental data
Meiothermus cerbereus DSM 11376(WP_027876468)	Unknown, needs experimental data
Meiothermus chliarophilus DSM 9957(WP_027893118)	Unknown, needs experimental data
Meiothermus ruber DSM 1279(AGK03951)	Unknown, needs experimental data
Meiothermus rufus DSM 22234(WP_027882714)	Unknown, needs experimental data
Meiothermussilvanus DSM 9946(ADH64647)	Unknown, needs experimental data
Meiothermus timidus DSM 17022(WP_018466224)	Unknown, needs experimental data
